# Beneficial Effects of Mineralocorticoid Receptor Antagonism on Myocardial Fibrosis in an Experimental Model of the Myxomatous Degeneration of the Mitral Valve

**DOI:** 10.3390/ijms21155372

**Published:** 2020-07-28

**Authors:** Jaime Ibarrola, Mattie Garaikoetxea, Amaia Garcia-Peña, Lara Matilla, Eva Jover, Benjamin Bonnard, Maria Cuesta, Amaya Fernández-Celis, Frederic Jaisser, Natalia López-Andrés

**Affiliations:** 1 Cardiovascular Translational Research, Navarrabiomed (Miguel Servet Foundation), Instituto de Investigación Sanitaria de Navarra (IdiSNA), 31008 Pamplona, Spain; jaime.ibarrola.u@gmail.com (J.I.); mgaraikoetx@alumni.unav.es (M.G.); amaiagpu@hotmail.com (A.G.-P.); lara.matilla.cuenca@navarra.es (L.M.); ej14025@bristol.ac.uk (E.J.); mcuesta.1@alumni.unav.es (M.C.); amaya.fernandez.decelis@navarra.es (A.F.-C.); 2 Centre de Recherche des Cordeliers, INSERM, Sorbonne Université, USPC, Université Paris Descartes, Université Paris Diderot, 75013 Paris, France; benjamin_bonnard@icloud.com (B.B.); frederic.jaisser@inserm.fr (F.J.); 3 Université de Lorraine, INSERM, Centre d’Investigations Cliniques-Plurithématique 1433, UMR 1116, CHRU de Nancy, French-Clinical Research Infrastructure Network (F-CRIN) INI-CRCT (Cardiovascular and Renal Clinical Trialists), Nancy, France

**Keywords:** myxomatous degeneration, cardiac fibrosis, mineralocorticoid receptor antagonist, collagen, proteoglycans

## Abstract

Mitral valve prolapse (MVP) patients develop myocardial fibrosis that is not solely explained by volume overload, but the pathophysiology has not been defined. Mineralocorticoid receptor antagonists (MRAs) improve cardiac function by decreasing cardiac fibrosis in other heart diseases. We examined the role of MRA in myocardial fibrosis associated with myxomatous degeneration of the mitral valve. Myocardial fibrosis has been analyzed in a mouse model of mitral valve myxomatous degeneration generated by pharmacological treatment with Nordexfenfluramine (NDF) in the presence of the MRA spironolactone. In vitro, adult human cardiac fibroblasts were treated with NDF and spironolactone. In an experimental mouse, MRA treatment reduced interstitial/perivascular fibrosis and collagen type I deposition. MRA administration blunted NDF-induced cardiac expression of vimentin and the profibrotic molecules galectin-3/cardiotrophin-1. In parallel, MRA blocked the increase in cardiac non-fibrillar proteins such as fibronectin, aggrecan, decorin, lumican and syndecan-4. The following effects are blocked by MRA: in vitro, in adult human cardiac fibroblasts, NDF-treatment-induced myofibroblast activation, collagen type I and proteoglycans secretion. Our findings demonstrate, for the first time, the contribution of the mineralocorticoid receptor (MR) to the development of myocardial fibrosis associated with mitral valve myxomatous degeneration. MRA could be a therapeutic approach to reduce myocardial fibrosis associated with MVP.

## 1. Introduction

Mitral valve prolapse (MVP) affects up to 2–3% of the general population, accounting for over 144 million individuals worldwide [1]. Fibromyxomatous degeneration of the mitral valve is generally acknowledged as the main etiological factor leading to MVP. The result is a redundant and abnormal thickening of the mitral valve leaflet that prolapses into the left atrium during systole. The most frequent complication of MVP is mitral regurgitation which may progress and cause heart failure (HF) [1]. Patients with chronic mitral regurgitation experience persistent volume overload, dilatation and enlargement of the left ventricle resulting in cardiac fibrosis [2,3,4]. Nevertheless, volume overload reported in patients with MVP is not sufficient to justify LV eccentric hypertrophy. Indeed, MVP patients show a higher degree of LV fibrosis not found in patients diagnosed with mitral regurgitation for other causes, suggesting different underlying pathogenesis despite similar clinical outcomes [5]. The presence of replacement fibrosis may lead to increased symptomatic ventricular arrhythmic events in patients with MVP [5]. Despite decades of investigations, the cellular and molecular mechanisms triggering myocardial fibrosis and mitral valve fibromyxomatous degeneration are yet to be fully understood.

Cardiac fibrosis refers to the accumulation of extracellular matrix (ECM) components in the myocardium, the cardiac fibroblast being the principal source of the ECM components [6]. Basic studies have shown that aberrant and perpetuated fibroblasts’ differentiation to myofibroblasts results in the excess deposition of ECM proteins [7]. The myofibroblasts exhibit a secretory phenotype and express activation markers such as α-smooth muscle actin (α-SMA) [8]. Therefore, the cardiac fibroblast is not able to maintain the ECM homeostasis. Instead, a continuous synthesis and degradation of ECM components are promoted and that is regulated by mechanical, electrical and neurohormonal stimulation [6,9]. ECM is composed by fibrillar proteins (such as collagen), and other non-fibrillar proteins (including fibronectin, proteoglycans, glycoproteins or glycosaminoglycans). The accumulation of both fibrillar and non-fibrillar proteins contributes to cardiac fibrosis [10]. One of the most studied fibrogenic axis is the Aldosterone/mineralocorticoid receptor (MR) pathway. Evidences from animal experiments in addition to the large randomized controlled trials RALES, EPHESUS and EMPHASIS studies in patients with HF suggest that chronic MR blockade consistently reduces the biological markers of cardiac fibrosis, suggesting that MR is an important determinant of cardiac collagen turnover [11,12,13,14].

Recently, our group has demonstrated for the first time that the MR pathway regulates mitral valve remodeling associated with mitral valve fibromyxomatous degeneration. Indeed, MR antagonist (MRA) treatment appears to be a promising option to reduce valve fibromyxomatous alterations associated to the development of MVP in animal models. Now we aim to investigate if MR signaling is also involved in the development of myocardial fibrosis associated with mitral valve fibromyxomatous degeneration and MVP progression by using in vivo and in vitro approaches.

## 2. Results

### 2.1. Effects of Spironolactone on Cardiac Fibrillar Proteins and Fibrotic Markers in a Murine Experimental Model of Fibromyxomatous Degeneration of the Mitral Valve

**Figure 1:**
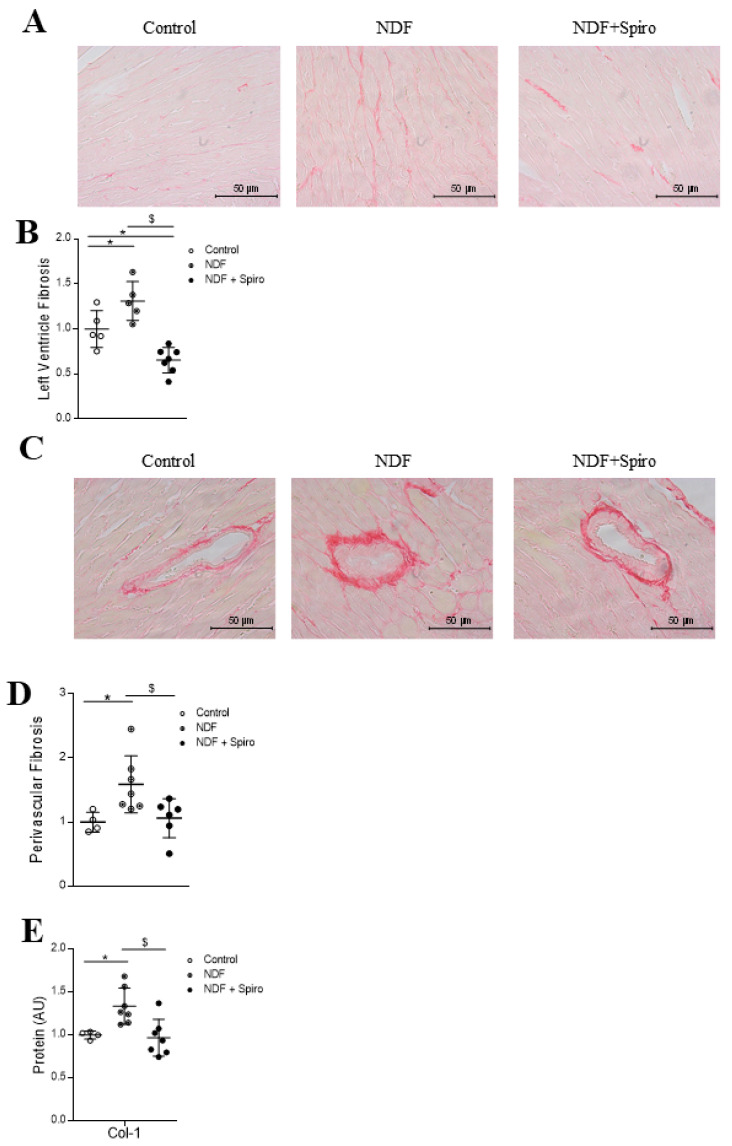
Effects of Spironolactone on cardiac fibrosis in a mouse model of myxomatous valve disease. Representative microphotographs of mouse myocardial sections with Sirius red staining for interstitial (**A**) and perivascular (**C**) fibrosis. Individual datapoints show the quantification of interstitial fibrosis (* *p* = 0.0452 Nordexfenfluramine (NDF) vs. control; ^$^ *p* = 0.0001; * *p* = 0.0166 NDF + Spiro vs. control) (**B**) and perivascular fibrosis (* *p* = 0.0307; ^$^ *p* = 0.035) (**D**). Quantification of collagen type I (* *p* = 0.0329; ^$^ *p* = 0.0007) (**E**). The box plots show the individual datapoints and the horizontal bars indicate the mean and SEM in arbitrary units versus the control group. Magnifications 40× (Scale bar 50 µm). The results were analyzed using one-way analysis of variance (ANOVA), followed by Tukey’s multiple comparisons tests. * vs. control, ^$^ vs. NDF.

Nordexfenfluramine (NDF)-treated mice presented a significant increment (*p* < 0.05) in LV interstitial fibrosis (Figure 1A,B) and perivascular fibrosis (Figure 1C,D) as compared to controls. NDF effect on both LV interstitial and perivascular fibrosis was blunted by spironolactone treatment (Figure 1B,D). The protein expression of collagen type I significantly increased (*p* < 0.05) in mice treated with NDF as compared to controls (Figure 1E). These effects were blocked by spironolactone treatment (*p* < 0.05) (Figure 1A–E).

**Figure 2:**
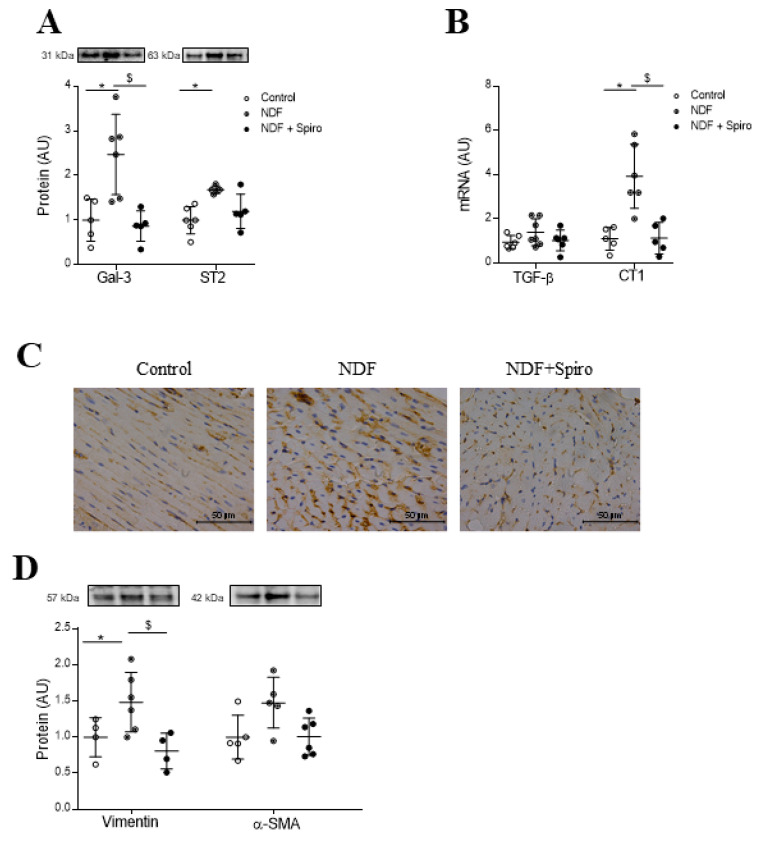
Effect of Spironolactone on profibrotic molecules and myofibroblast activation markers in a mouse model of myxomatous mitral valve disease. Quantification of Gal-3 (* *p* = 0.0338; ^$^ *p* = 0.0217) and ST2 (* *p* = 0.0289) (**A**) protein levels in myocardium from controls, NDF-treated mice and NDF + Spiro-treated mice. Quantification of TGF-β (Transforming growth factor beta) and CT-1 (Cardiotrophin-1) (* *p* = 0.0014; ^$^ *p* = 0.0016) (**B**) mRNA levels from controls, NDF-treated mice and NDF + Spiro-treated mice. Representative microphotographs of α-smooth muscle actin (α-SMA) immunostaining in myocardium from controls, NDF-treated mice and NDF + Spiro-treated mice (**C**). Quantification of vimentin (* *p* = 0.0033; ^$^ *p* = 0.0051) and α-SMA protein levels in myocardium from controls, NDF-treated mice and NDF +Spiro-treated mice (**D**). The box plots show the individual datapoints and the horizontal bars indicate the mean and SEM in arbitrary units versus the control group. Magnifications 40× (Scale bar 50 µm). The results were analyzed using one-way ANOVA, followed by Tukey’s multiple comparisons tests. * vs. control, ^$^ vs. NDF.

The expression of the fibrotic markers Gal-3 and ST-2 was also enhanced (*p* < 0.05) by NDF treatment, although only Gal-3 synthesis induced by NDF was blocked (*p* < 0.05) by spironolactone (Figure 2A). Regarding other profibrotic markers, NDF-treated mice presented similar mRNA levels of TGF-β and increased (*p* < 0.05) levels of cardiotrophin-1 (CT-1) (Figure 2B). MRA blocked (*p* < 0.05) the elevated levels of CT-1 mRNA (Figure 2B). The increases in β-SMA immunostaining and in vimentin protein expression (*p* < 0.05) in NDF mice were fully prevented by spironolactone treatment (Figure 2C,D). See the original Western blot images in Supplemental Figure S1.

### 2.2. Effects of Spironolactone on Cardiac Non-Fibrillar Proteins in A Murine Experimental Model of the Fibromyxomatous Degeneration of the Mitral Valve

**Figure 3:**
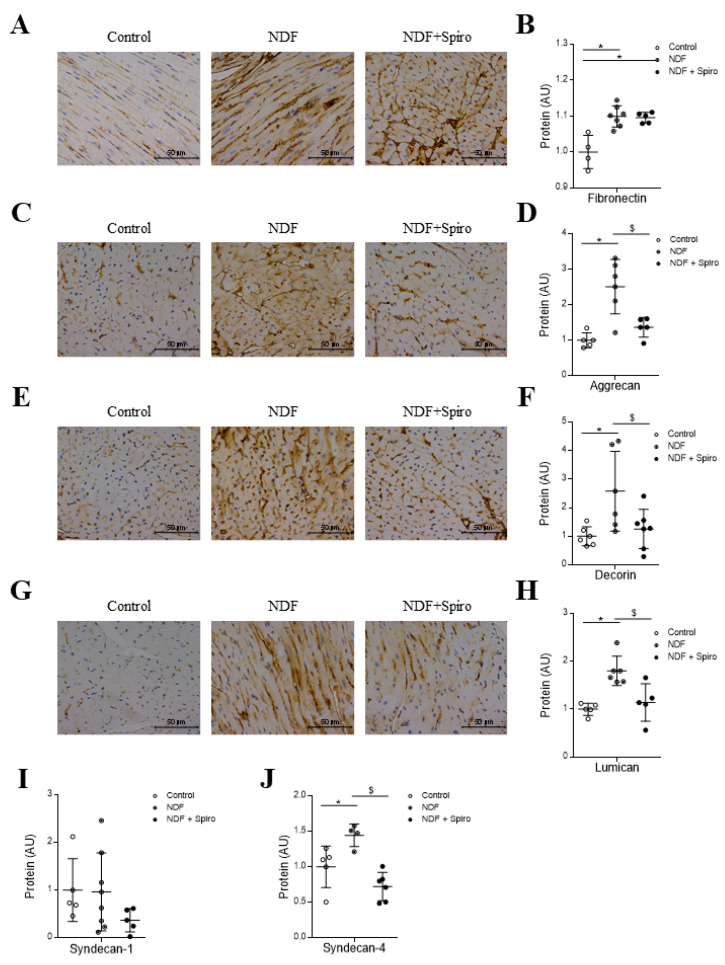
Effect of spironolactone treatment on the expression of non-fibrillar proteins in a mouse model of mitral valve fibromyxomatous degeneration. Representative microphotographs of fibronectin, aggrecan, decorin and lumican immunostainings are shown (**A**,**C**,**E**,**G**). The protein expressions of fibronectin (* *p* = 0.0005 Control vs. NDF; * *p* = 0.0013 Control vs. NDF+Spiro), aggrecan (* *p* = 0.0008; ^$^ *p* = 0.0072), decorin (* *p* = 0.0209; ^$^ *p* = 0.0459), lumican (* *p* = 0.0017; ^$^ *p* = 0.0072), syndecan-1 and syndecan-4 (* *p* = 0.032; ^$^ *p* = 0.0009) were measured by ELISA (Enzyme-Linked Immuno Sorbent Assay) in myocardium from controls, NDF-treated mice and NDF +Spiro-treated mice (**B**,**D**,**F**,**H**–**J**). The box plots show the individual datapoints and the horizontal bars indicate the mean and SEM in arbitrary units versus the control group. Magnifications 40× (Scale bar 50 µm). The results were analyzed one-way ANOVA, followed by Tukey’s multiple comparisons tests. * vs. control, ^$^ vs. NDF.

NDF-treated mice exhibited higher levels (*p* < 0.05) of other non-fibrillar proteins, such as fibronectin (Figure 3A,B), aggrecan (Figure 3C,D), decorin (Figure 3E,F), and lumican (Figure 3G,H) as compared to controls (*p* < 0.05). In spironolactone-treated mice, proteoglycans expression and immunostainings were normalized as compared with NDF-treated mice (*p* < 0.05) (Figure 3A–H). Fibronectin secretion was increased in spironolactone-treated mice as compared with the control group (*p* < 0.05) (Figure 3B). Concerning the expression of the surface membrane proteoglycans, NDF treatment did not modify syndecan-1 levels but increased (*p* < 0.05) syndecan-4 expression (Figure 3I,J), this effect being prevented by Spironolactone treatment (*p* < 0.05) (Figure 3J).

### 2.3. Mineralocorticoid Receptor Mediates the Profibrotic Response of Human Cardiac Fibroblasts to NDF

**Figure 4:**
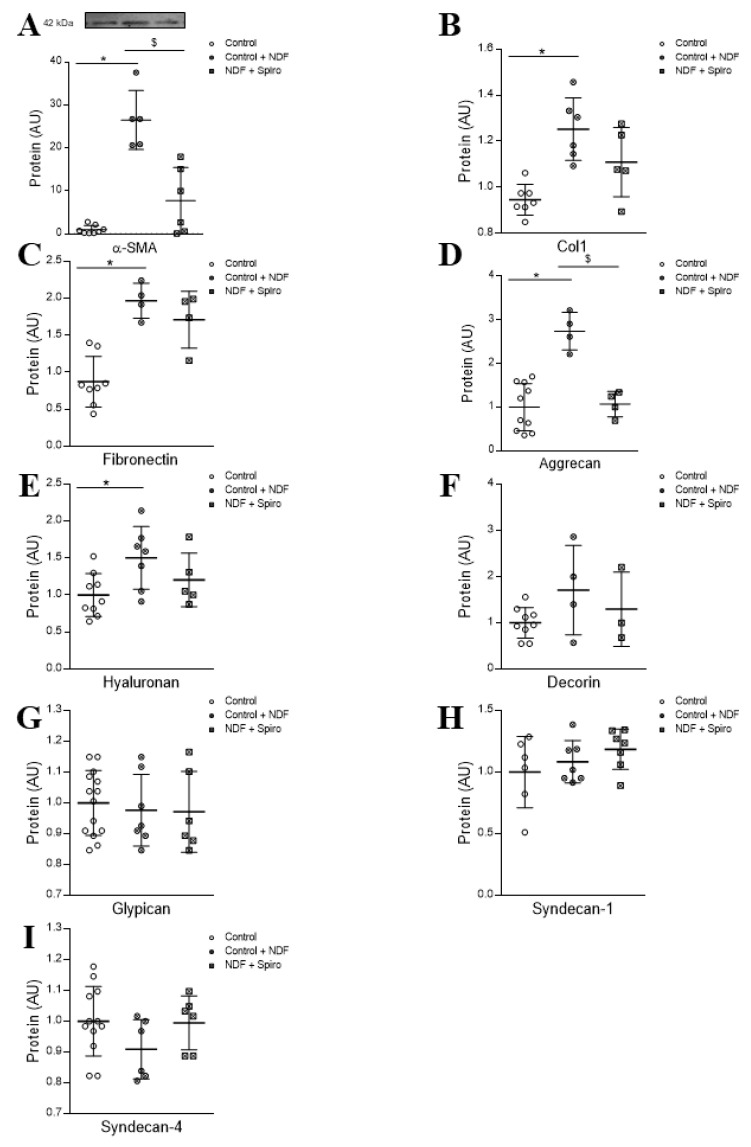
NDF induces the expression of activation and fibrosis markers in adult human cardiac fibroblast. NDF effects on the activation marker α-SMA protein expression in adult human cardiac fibroblasts (* *p* = 1.0 × 10^−7^; ^$^ *p* = 1.6 × 10^−5^ Control + NDF vs. NDF + Spiro) (**A**). Quantification of collagen type I (* *p* = 0.0410; ^$^ *p* = 0.0208 (**B**) and the non-fibrillar protein fibronectin (* *p* = 0.0172)) (**C**) in human cardiac fibroblasts treated with NDF and NDF+Spiro. Quantification of proteoglycans (aggrecan (* *p* = 6 × 10^−6^; ^$^ *p* = 1.25 × 10^−4^ Control+NDF vs. NDF+Spiro) (**D**), hyaluronan (**E**), decorin (**F**), glypican (**G**), syndecan-1 (**H**) and syndecan-4 (**I**) in human cardiac fibroblasts treated with NDF and NDF + Spiro. The box plots show the individual datapoints and the horizontal bars indicate the mean and SEM in arbitrary units versus the control group. The results were analyzed using one-way ANOVA, followed by Tukey’s multiple comparisons tests. * vs. control, ^$^ vs. control +NDF.

NDF-treated human cardiac fibroblasts presented an increment (*p* < 0.05) in the activation marker α-SMA (Figure 4A). This expression was mitigated (*p* < 0.05) when the fibroblasts were treated with the MR inhibitor spironolactone (Figure 4A). Collagen type I and fibronectin secretion were increased by NDF treatment (*p* < 0.05) (Figure 4B,C). NDF treatment augmented aggrecan and hyaluronan secretions (*p* < 0.05) (Figure 4D,E) without modifying decorin, glypican, syndecan-1 or syndecan-4 levels (Figure 4F–I). NDF-dependent upregulation of aggrecan was prevented by spironolactone (*p* < 0.05) (Figure 4D) reaching levels close to those for controls. Hyaluronan was upregulated by NDF treatment (*p* < 0.05) (Figure 4E). See the original Western blot images in Supplemental Figure S1.

## 3. Discussion

The goal of this study was to investigate myocardial ECM changes associated with the development of fibromyxomatous mitral valve alterations. Our results demonstrate that the experimental model of mitral valve fibromyxomatous disease presents myocardial fibrosis. The latter was demonstrated by increased fibrillar ECM, mainly collagen type I, as well as nonfibrillar proteins, including fibronectin, extracellular proteoglycans, small leucine-rich proteoglycans and surface membrane proteoglycans. MR blockade exerts beneficial effects by preventing cardiac ECM alterations associated with mitral valve fibromyxomatous degeneration that could ultimately lead to MVP. Moreover, the following effects are partially blocked by an MR antagonist: in vitro, NDF treatment induces myofibroblast activation, collagen secretion and increases the expression of some non-fibrillar ECM proteins.

NDF belongs to a group of anorectic compounds that have been associated with the remodeling of mitral and aortic valves [15]. These drugs interact with the serotonergic system by targeting the Serotonin (5-HT) receptor subtypes [16,17]. Besides, several studies demonstrated that these drugs and the activation of the 5-HT pathway not only induce valve remodeling but also cardiac fibrosis altering the myocardial ECM composition [18,19,20]. The effect of these drugs induces a loss of cardiac ECM homeostasis, accumulation of interstitial fibroblasts and collagen deposition [19,21,22,23]. Our results in vitro, in human cardiac fibroblasts, and in vivo, in NDF-treated mice, are in line with these pieces of evidence. In a mitral valve, a fibromyxomatous degeneration mice model previously characterized [24,25] cardiac interstitial and perivascular fibrosis were enhanced. Furthermore, NDF treatment induced cardiac fibroblasts’ activation and an increase in fibrillar and non-fibrillar ECM proteins, as well as the fibrosis markers Gal-3, ST2 and CT-1. Interestingly, the use of MRA prevented interstitial and perivascular fibrosis as well as the increase in fibrillar and non-fibrillar ECM proteins. However, in vitro spironolactone did not block the increase in all the fibrosis markers induced by NDF, including fibronectin synthesis. Of interest, only one dose of spironolactone has been used, and we cannot exclude the possibility that other doses could exert an effect on these markers. MR has a well-established pathophysiological role in cardiovascular diseases [26]. Although this is the first time that an MRA has been tested in the context of cardiac ECM changes associated with mitral valve fibromyxomatous alterations, the use of MRA as anti-fibrotic has been tested in other cardiovascular diseases [11,27,28,29].

Our study provides information about the role of the non-fibrillar proteins in myocardial fibrosis. The extracellular proteoglycans such as aggrecan, the small leucine-rich proteoglycans, including decorin and lumican, and the cell surface proteoglycan syndecan-4 [10,30] were the principal proteoglycans altered in cardiac fibrosis associated with mitral valve fibromyxomatous degeneration that could lead to MVP. The extracellular proteoglycans are the main proteoglycans involved in the stabilization of the ECM and collagen synthesis [10]. Decorin and lumican are increased in myocardial fibrosis following pressure overload or myocardial infarction [31], while syndecan-4 contributes to myofibroblast differentiation in these settings [32]. Interestingly, the MR pathway is involved in the upregulation of all these proteoglycans in vitro and in vivo in an experimental model of NDF-induced mitral valve fibromyxomatous alterations. It has been shown that aldosterone, via MR, induces ADAMTS1, the enzyme that degrade aggrecan [33]. Moreover, MRA also blunted the increase in proteoglycans and showed in the mitral valves of NDF-treated mice [25]. However, this is the first time that the expression of cardiac proteoglycans is analyzed in an experimental model of fibrosis associated with myxomatous mitral valve disease. Further studies are warranted to unravel the specific role of non-fibrillar proteins in myocardial fibrosis as well as the benefits of an MRA therapy.

Cardiac fibrosis in MVP has been classically considered secondary to volume overload [34,35]. However, recent studies have shown higher degree of myocardial fibrosis in MVP patients compared to patients with mitral regurgitation due to other etiologies, regardless of the severity of mitral regurgitation or cardiac remodeling [5,36,37,38]. On the other hand, MVP is associated with an increased rate of ventricular arrhythmias and sudden cardiac death [5,39], which, in turn, has been associated with the presence of myocardial fibrosis in these patients [5,40]. It has been suggested that myocardial fibrosis in this scenario may occur as a consequence and a response to recurring mechanical stretching [41]. Thus, therapies targeting LV fibrosis in the context of MVP are needed. Future clinical studies are needed to analyze the influence of antifibrotic therapies such as MRA in MVP.

## 4. Materials and Methods

### 4.1. In Vivo Studies

Ten-week old male wild-type 129S2/Sv mice (Charles River Laboratories) were used in order to reproduce the model used by Monassier and co-workers [24]. Osmotic minipumps (Alzet) delivering Nordexfenfluramine (NDF) (1 mg/kg/day; Sigma-Aldrich, Sigma/Merck Life Sciences S.L.U., Madrid, Spain) were implanted subcutaneously. The MRA Spironolactone (1 mg/kg per day) was administered as an additive in the food for 28 days. Animals were housed in a climate-controlled facility with a 12 h/12 h light/dark cycle. The experiments were approved (1 June 2017) by the Darwin ethics committee of Pierre et Marie Curie University and conducted according to the INSERM (Institut national de la santé et de la recherche médicale) animal care and use committee guidelines (APAFIS#4488-20 1 6010614517136 v3).

### 4.2. Cell Culture

Human cardiac fibroblasts were obtained from Promocell and maintained in medium Fibroblasts Media 3. The cells were cultured according to the manufacturer’s instructions. The cells were used between passages 4 and 6. The cells were stimulated with NDF (10^−5^ M, Sigma-Aldrich) for 24 h (the concentration was chosen based on the literature) [21]. The MR antagonist spironolactone (Spiro, 10^−6^ M, Sigma-Aldrich) was added for 30 min prior to the stimulation with NDF.

### 4.3. Real-Time Reverse Transcription PCR

Total RNA was extracted with Trizol Reagent (Qiagen), according to the manufacturer’s instructions. First-strand cDNA was synthesized according to the manufacturer’s instructions (Bio-Rad, Hercules, CA, USA). Quantitative PCR analysis was performed with SYBR green PCR technology (Bio-Rad) (Supplemental Table S1) according to the following PCR conditions: Initial Denaturation; the reaction temperature is increased to 95 °C and incubated for 2 min to ensure that all complex, double-stranded DNA (dsDNA) molecules are separated into single strands for amplification. Cycling: (1) Denaturation: The reaction temperature is increased to 95 °C, which melts (disrupts the hydrogen bonds between complementary bases) all dsDNA into single-stranded DNA (ssDNA) (10 s); (2) Annealing: The temperature is lowered to approximately 5 °C below the melting temperature (Tm) of the primers (60 °C) to promote primer binding to the template (30 s); (3) Extension: The temperature is increased to 72 °C, which is the optimum for DNA polymerase activity to allow the hybridized primers to be extended (30 s); Repeat: Steps 1–3 are performed in a cyclical manner, resulting in the exponential amplification of the amplicon. Relative quantification was achieved with MyiQ software. The data were normalized by HPRT, GADPH and β-actin levels and expressed as percentage relative to controls. All PCRs were performed at least in triplicate for each experimental condition.

### 4.4. Western Blot Analysis

Aliquots of 20 µg of total proteins were prepared from cell extracts or cardiac homogenates and electrophoresed on SDS polyacrylamide gels and transferred to Hybond-c Extra nitrocellulose membranes (Bio-Rad). Membranes were incubated with primary antibodies for: vimentin (Sigma), α-Smooth Muscle Actin (α-SMA; Sigma), galectin-3 (Gal-3; Santa Cruz, CA, USA), ST2 (Novus Biologicals, Centennial, CO, USA). Stain-free detection was used as a loading control. After washing, detection was made through incubation with peroxidase-conjugated secondary antibody and developed using an ECL (enhanced luminol-based chemiluminescent) chemiluminescence kit (Amersham, GE healthcare, Thermo Fisher Scientific, UK). After densitometric analyses, optical density values were expressed as arbitrary units. All Western blots were performed at least in triplicate for each experimental condition.

### 4.5. Immunohistological Evaluation

Histological determinations in mouse cardiac tissue were performed in 5-μm-thick sections. The immunochemistry was performed following the protocol of Leica BOND-Polymer Refine Detection automatic immunostainer (Leica). All solutions were filled into the bottle-Bond Open Container (Leica) and registered on computer using the Leica Biosystem program. The immunostaining program protocol include: fixative solution, bond wash solution, blocking with common immunohistochemistry blocker and incubated with the primary antibody for α-SMA (Sigma), fibronectin (Santa Cruz), decorin (Santa Cruz), lumican (Abcam, Cambridge, UK), aggrecan (Abcam). After primary antibody incubation, the slides were incubated with post primary poly-HRP-IgG. The signal was revealed by using DAB (3,3′-Diaminobenzidine) Substrate. As negative controls, samples followed the same procedure described above but were used in the absence of primary antibodies. For Sirius red staining, slides were hydrated and incubated with 1% Sirius red in picric acid for and 30 min. For each immunochemistry and staining, serial sections were done and quantified. In the figures, the most representative image of each experimental condition is shown.

### 4.6. ELISA

Collagen type I, fibronectin, decorin, lumican, aggrecan, hyaluronan, syndecan-1, syndecan-4 and glypican were measured in cardiac homogenates and cell supernatants by ELISA according to the manufacturer’s instructions (R&D Systems).

### 4.7. Statistical Analyses

For the in vivo study, the data were expressed as mean ± SD. The normality of distributions was verified by means of the Lilliefors-corrected Kolmogorov–Smirnov test. The data were analyzed using a one-way analysis of variance (ANOVA) followed by a Tukey’s tests to assess specific differences among groups or conditions using GraphPad Software Inc. For animal studies, the sample size calculation software G Power (http://www.gpower.hhu.de/, last access date: 1st February 2020) was used. The sample size of each experiment was determined by power analyses based on data from previous studies and preliminary experiments with alpha of 0.05 and 85% power.

For the in vitro experiments, data were expressed as mean ± SD. The normality of distributions was verified by means of the Lilliefors-corrected Kolmogorov–Smirnov test. The data were analyzed using Student’s test. A *p* value of < 0.05 was considered significant.

## 5. Conclusions

**Figure 5:**
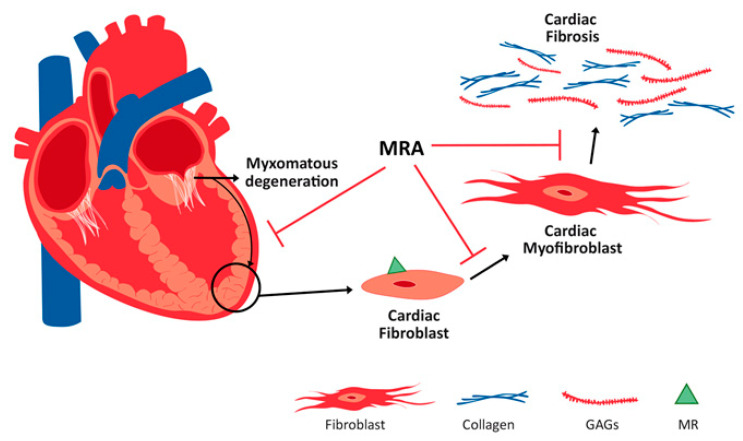
Refining the mineralocorticoid receptor antagonist (MRA) as a new pharmacological approach to treat myocardial fibrosis associated with myxomatous mitral valve disease.

In conclusion, in an experimental model of myxomatous mitral valve disease, myocardial ECM remodeling and fibrosis are mediated by MR. Our results suggest that MRA could be a therapeutic approach to reduce myocardial fibrosis associated with MVP (Figure 5).

### 5.1. Translational Perspective

Patients with mitral valve prolapse develop myocardial fibrosis, although the cellular and molecular mechanisms are not known. We have characterized myocardial fibrosis in an experimental model of fibromyxomatous degeneration of the mitral valve. Moreover, our results suggest that mineralocorticoid receptor antagonism could exert beneficial effects by reducing myocardial fibrosis associated to mitral valve fibromyxomatous disease.

### 5.2. Limitations

This study had several limitations. First, all animal studies were done in males. Second, cardiac and valvular function was not evaluated in the animal models.

## Supplementary Materials

The following are available online at https://www.mdpi.com/1422-0067/21/15/5372/s1.

## Abbreviations

MVP	Mitral valve prolapse
NDF	Nordexfenfluramine
MR	Mineralocorticoid receptor
MRA	Mineralocorticoid receptor antagonist
ECM	Extracellular matrix
HF	Heart failure
