# Supplementary material for: Beneficial Effects of Mineralocorticoid Receptor Antagonism on Myocardial Fibrosis in an Experimental Model of the Myxomatous Degeneration of the Mitral Valve
Source: Int J Mol Sci. 2020 Jul 28;21(15):5372. doi: 10.3390/ijms21155372 (PMC7432373; doi:10.3390/ijms21155372)

Full unedited gel for Figure 2A

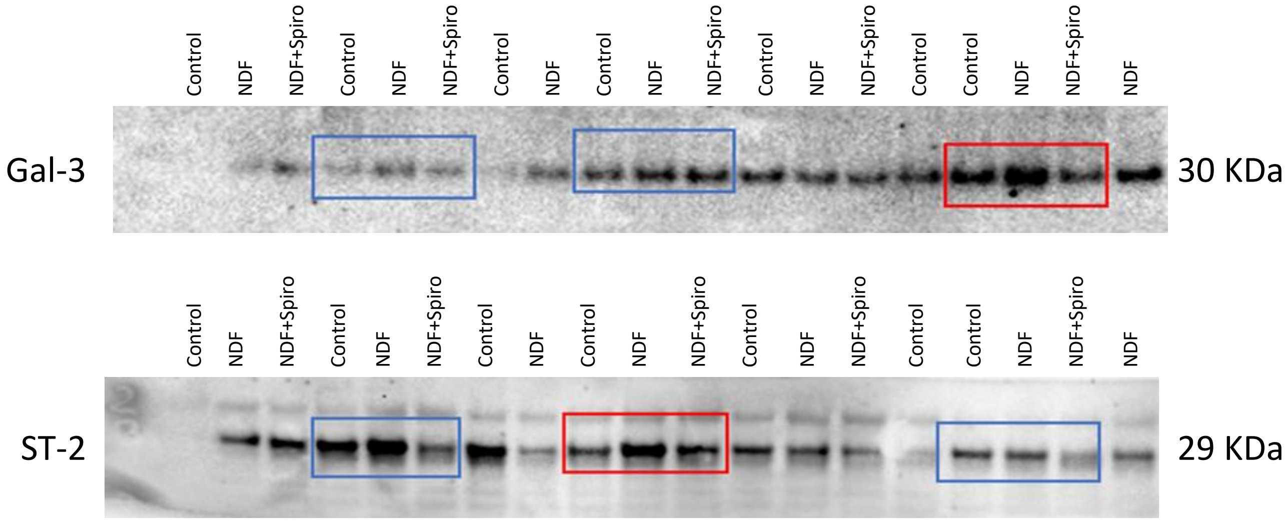

Full unedited gel for Figure 2D

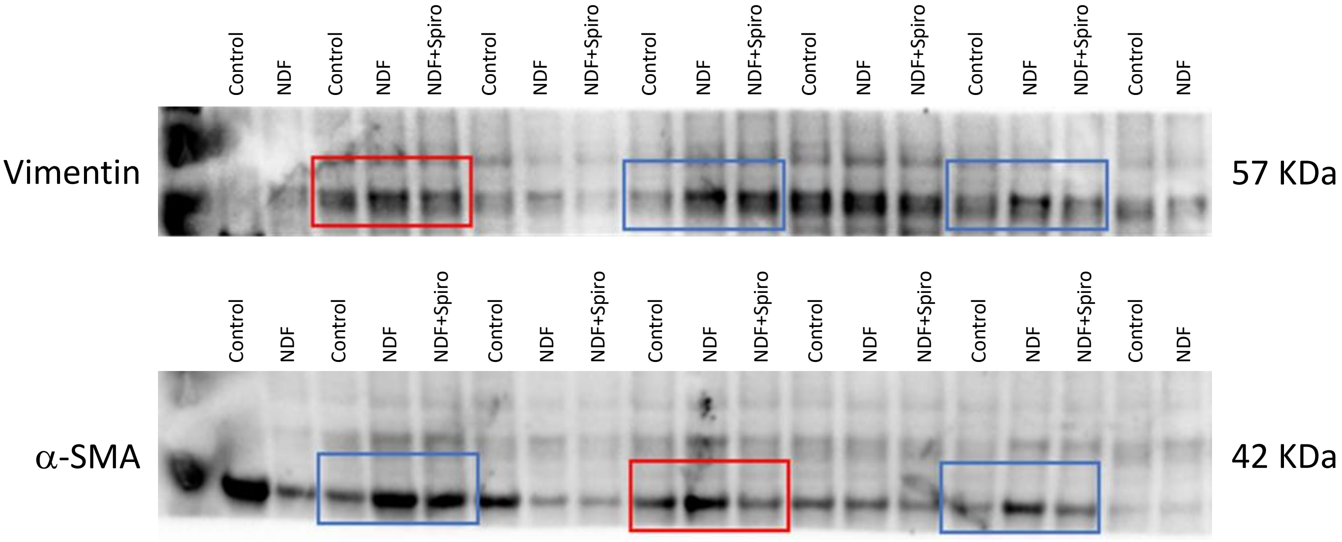

Full unedited gel for Figure 4A

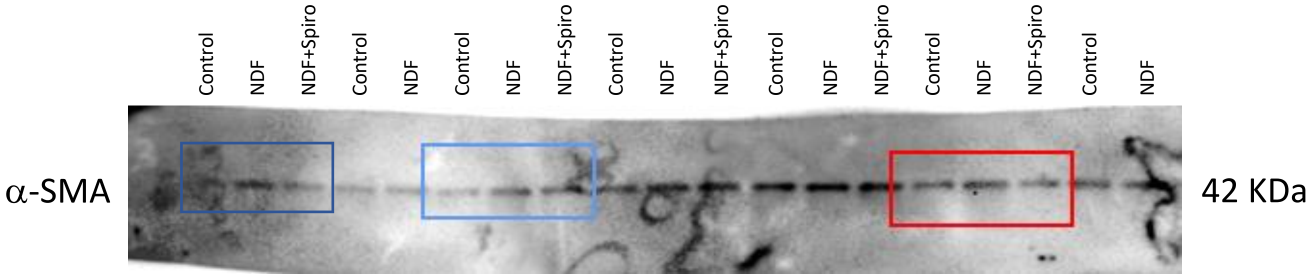

Stain free gel for Figure 2A

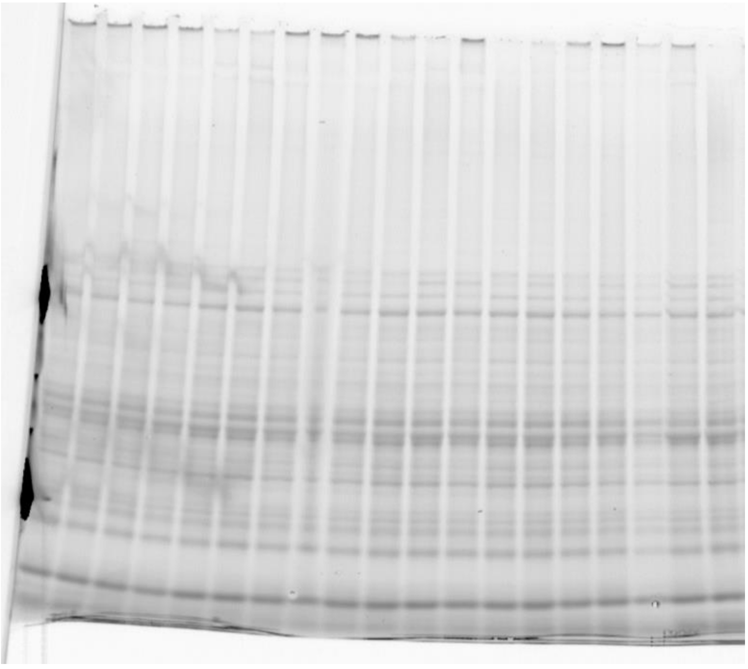

Stain free gel for Figure 2D

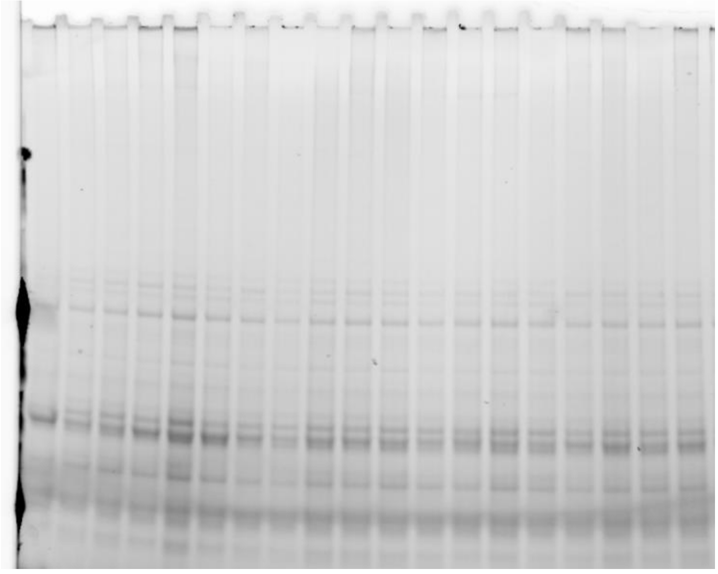

Stain free gel for Figure 4A

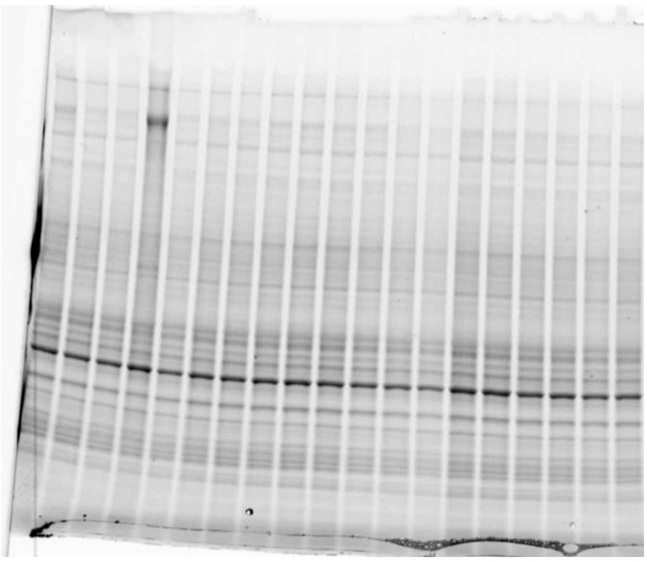

Supplement: Supplementary file 1 [file ijms-21-05372-s001.zip › ijms-863715 Supplemental Figure S1 - Primers used in real time PCR analysis.pdf]
