# Supplementary material for: Beneficial Effects of Mineralocorticoid Receptor Antagonism on Myocardial Fibrosis in an Experimental Model of the Myxomatous Degeneration of the Mitral Valve
Source: Int J Mol Sci. 2020 Jul 28;21(15):5372. doi: 10.3390/ijms21155372 (PMC7432373; doi:10.3390/ijms21155372)
Supplement: Supplementary file 1 [file ijms-21-05372-s001.zip › ijms-863715 Supplemental Table S1 .pdf]

SUPPLEMENTAL TABLE

Table S1: Primers used in humans in real time PCR analysis

**Gene**	**Primer**	**Sequence (5´to 3´)**
**TGF-β**	Forward	GAC GTC ACT GGA GTT GTA CGG
	Reverse	GCT GAA TCG AAA GCC CTG T
**CT-1**	Forward	TCA TTC CTA CCC CAT TTG GA
	Reverse	ACA CCG GTA GCC CTG CAT
**Gal-3**	Forward	CAG TGC TCC TGG AGG CTA TC
	Reverse	ATT GAA GCG GGG GTT AAA GT
